# Visual outcomes of proton beam therapy for choroidal melanoma at a single institute in the Republic of Korea

**DOI:** 10.1371/journal.pone.0242966

**Published:** 2020-12-02

**Authors:** Su-Kyung Jung, Young-Hoon Park, Dong-ho Shin, Hak-Soo Kim, Jong-Hwi Jung, Tae-Hyun Kim, Sung Ho Moon

**Affiliations:** 1 Department of Ophthalmology, Hospital, National Cancer center, Gyeonggi-do, Korea; 2 Department of Ophthalmology, Seoul St. Mary’s Hospital, The Catholic University of Korea, College of Medicine, Seoul, Korea; 3 Proton Therapy Center, Research Institute and Hospital, National Cancer center, Gyeonggi-do, Korea; Medical Research Foundation (Sankara Nethralaya), INDIA

## Abstract

We evaluate the ocular effects of proton beam therapy (PBT) in a single institution, in Korea, and identify factors contributing to decreasing visual acuity (VA) after PBT. A total of 40 patients who received PBT for choroidal melanoma (2009‒2016) were reviewed. Dose fractionation was 60‒70 cobalt gray equivalents (CGEs) over five fractions. Complete ophthalmic examinations including funduscopy and ultrasonography were performed at baseline and at 3, 6, and 12 months after PBT, then annually thereafter. Only patients with at least 12 months follow-up were included. During the follow-up, consecutive best-corrected visual acuity (BCVA) changes were determined, and univariate and multivariate logistic regression analyses were performed to identify predictors for VA loss. The median follow-up duration was 32 months (range: 12‒82 months). The final BCVA of nine patients was > 20/40. The main cause of vision loss was intraocular bleeding, such as neovascular glaucoma or retinal hemorrhage. Vision loss was correlated with the tumor size, tumor distance to the optic disc or fovea, maculae receiving 30 CGEs, optic discs receiving 30 CGEs, and retinas receiving 30 CGEs. Approximately one-third of PBT-treated choroidal melanoma patients with good pretreatment BCVA maintained their VA. The patients who finally lost vision (VA < count fingers) usually experienced rapid declines in VA from 6‒12 months after PBT. Tumor size, tumor distance to the optic disc or fovea, volume of the macula, and optic discs or retinas receiving 30 CGEs affected the final VA.

## Introduction

Uveal melanoma (UM) is the most common primary intraocular malignancy. It is well known that the incidence of UM in Western countries is higher than that in Asian countries [1, 2]. The annual incidence of UM in Asia is 0.25 cases per one million individuals, but the age-standardized incidence rate increased recently to 0.60 cases per one million individuals [3, 4]. Because of the limited number of cases, there have been a few studies reporting treatment outcomes, toxicity, or prognoses of UM in Asia [5–8].

Eyeball-conserving treatments such as proton beam therapy (PBT), helium, and carbon, and plaque brachytherapy have been accepted as standard first-line treatments for localized UM [9, 10]. Our previous study showed that the local control rate and complication profile after PBT in patients with UM in the Republic of Korea were comparable with those in previous PBT series [11]. The ideal goals of radiation therapy are to maintain the visual function and effectively control local tumors. However, it induces capillary closure, telangiectasia, microaneurysm formation, hemorrhage, exudates, macular edema, and nerve fiber layer infarctions, and can ultimately result in a poor visual outcome [12]. There are several studies identifying predictors of final visual acuity (VA) after PBT in Western countries [13, 14]. The ocular effects of PBT are expected to differ by race, considering the diversity in the amounts of uveal tissue pigments present among different races. However, there have been few studies investigating the factors affecting visual outcomes after PBT in Asia.

In this study, we reviewed the visual outcomes and ocular effects in patients who received PBT for UM in a single institution in Asia, and also identified the factors affecting VA after PBT.

## Methods

The institutional review board of the National Cancer Centre approved this study (NCC2020-0054), which was performed according to the principles of the Declaration of Helsinki. We performed a retrospective chart review of all patients who received PBT for UM between 2009 and 2016, and a total of 40 were included in this study. The selection criteria for PBT were as follows: no distant metastasis, tumor diameter ≤ 24 mm, tumor height ≤ 14 mm, tumor volume < 30% of the ocular volume, and no full retinal detachment [11]. Ophthalmic work-up consisting of a slit lamp examination, fundus examination including fundus photography, ultrasonography (USG), and orbit magnetic resonance image (MRI) to diagnose the UM. Systemic work-up of computed tomography (CT) of the liver, or 18-fluorodeoxyglucose positron emission tomography were performed to check the distant metastasis.

As preparation for PBT, tantalum markers were fixated to demarcate the tumor boundary, and thin slice (0.625 mm) planning CT images were obtained with the patient in the supine position gazing straight forward. In addition, orthogonal pairs of orbital X-ray radiographs were captured with the patient in the sitting position on a dedicated chair using a bite-block and facial mask while maintaining a straight gaze during simulation. The target volume delineation for PBT was modeled using the analytic function in EYEPLAN (Varian Medical Systems, Palo Alto, CA, USA) to ensure that it closely matched the panoramic details in fundoscopic images, pretreatment MRI, and reconstructed thin slice CT images. Static tantalum clip positions were reconstructed on the eye globe as close to the simulation positions as possible. In the planning stage, a gaze direction was selected along the optimal treatment setup to deliver the proton beam and minimize the dose to the optic disc, macula, anterior structures (lens, cornea, and anterior chamber), lacrimal gland, and eyelids. In the verification stage, the clip positions of the treatment gaze angle were matched with those acquired from the EYEPLAN system. A brass aperture was then manufactured with a 3 mm margin around the tumor. The dose prescription was 60‒70 cobalt gray equivalents (CGEs) in five fractions [15, 16].

Patients were routinely followed-up at 1 month after PBT for acute reactions and every 3 months thereafter. During the follow-up period, complete ophthalmic examinations including best-corrected visual acuity (BCVA) were performed at every visit. Follow-up orbital MRIs including USG were performed at 3, 6, and 12 months after PBT and annually thereafter for most patients to determine the local progression exactly and evaluate posterior segment of eyeball. Only patients with at least 12 months follow-up were included in this study. VA decline including local recurrence, distant metastasis, enucleation, intraocular hemorrhage incidences was observed with Kaplan–Meier analysis. And pretreatment BCVA ≥ 20/40 was selected to find the prognostic factors affecting VA. After PBT, BCVA in the treated eye was classified as follows: ≥ 20/50 and 20/64 or worse. Comparisons between groups were performed using the Mann-Whitney U-test. Univariate and forward stepwise multivariate logistic regression analyses including correlation analysis were performed to identify predictors for loss of VA among patients, and tumor and dose-volume histogram (DVH) parameters. Significance was defined as a value of *p* < 0.05. Statistical evaluations were performed using the Statistical Package for the Social Sciences, version 18.0 (SPSS, Chicago, IL, USA).

## Results

In total, 40 patients were included in this study (Table 1). The median age was 56.5 years (range: 36‒87 years), and patients > 60 years of age constituted 42.5% of the total population. A total of 23 patients (57.5%) were female. The median tumor height was 7.0 mm, and the largest median tumor diameter was 17.42 mm. The overall median follow-up period was 32 months (range: 12‒82 months).

**Table 1:** Characteristics of patient treated with proton beam radiation therapy (PBRT).

Variable	Number
Age (years)	
Median age (range)	56.5 (36–87)
≤60	23
>60	17
Gender	
Male	17
Female	23
Involved eye	
Right	20
Left	20
Tumor height (mm), median (range)	7.0 (1.0–12.0)
Largest tumor diameter (mm), median (range)	17.42 (6.31–21.54)
Tumor distance to fovea (mm), median (range)	5.3 (0.63–17.1)
Tumor distance to optic disc edge (mm), median (range)	5.9 (0.18–19.9)
Involving ciliary body cases (%)	3 (7.5%)
Cases with diabetes at diagnosis	5 (12.5%)
Follow- up (months), median (range)	32 (12–82)

**Fig 1:**
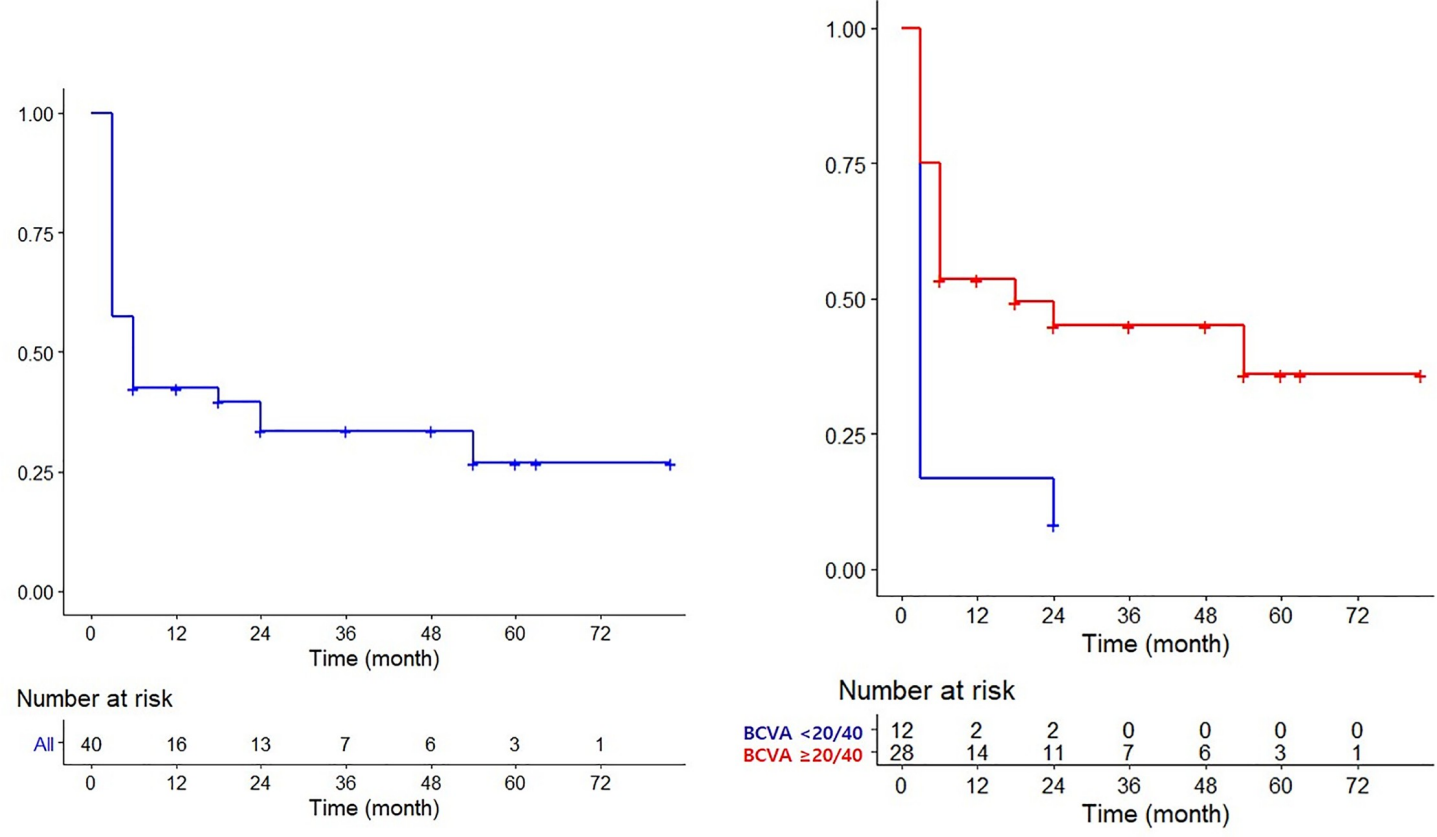
Kaplan-Meier Curve of vision loss after Proton Beam Therapy for all choroidal melanoma patients (a) and for choroidal melanoma patients according to the pretreatment BCVA (b).

Fig 1 shows the Kaplan Meier curve of the vision after PBT in choroidal melanoma patients. We defined that vision loss was final BCVA was under 20/80. Fig 1A showed the vision loss trend of all patients (N = 40) during the follow-up period, Zero meant that BCVA was under 20/80 and vision loss occurred. The 1-year, 3-year and 5 year vision survival probability was 0.425, 0.336 and 0.269. Fig 1B showed the different trends according to the pretreatment BCVA. The patients who had pretreatment BCVA <20/40 (N = 12, blue line) experienced more rapid visual decrease than the patients who had pretreatment BCVA ≥20/40 (N = 28, red line). Most patients who had pretreatment BCVA <20/40 abruptly got the poor VA after PBT. For this reason, the analysis for prediction factors of final BCVA was done in the patient who had pretreatment BCVA ≥20/40.

Twenty-eight patients had pretreatment BCVA ≥ 20/40 and the reminder (n = 12) had pretreatment BCVA < 20/40. The clinical features of patients who had pretreatment BCVA ≥ 20/40 were compared according to the final BCVA after PBT in Table 2. Twenty-eight patients who had pretreatment BCVA ≥ 20/40 were divided into patients who had a final BCVA ≥ 20/50 and those who had a final BCVA < 20/50 during the total follow-up period after PBT, respectively. The number in each group was 14. We compared the possible factors expected to affect the final BCVA between these two groups. The largest tumor diameter or tumor height reflecting the tumor size was larger in the final BCVA < 20/50 group than in the final BCVA ≥ 20/50 group after PBT, and these differences were statistically significant. Radiation dose exposure to the retina, macula, or optic disc in the final BCVA < 20/50 group was significantly higher than in the final BCVA ≥ 20/50 group (*p* < 0.05). However, radiation dose delivered to the ciliary body or lens did not show differences between these two groups.

**Table 2:** Clinical features of patients who had pretreatment BCVA (≥20/40) and finally get the BCVA (≥20/50) or not.

	Final BCVA ≥20/50 (No of patients = 14)	Final BCVA < 20/50 (No. of patients = 14)	p*
Gender			
Male	7	7	1.0
Female	7	7	
Age (years)			
median (range)	55(36–87)	54(45–79)	0.84
Tumor height (mm),	5.9	8.0	0.01
Largest tumor diameter (mm),	14.6	17.4	0.02
Tumor distance to fovea (mm),	7.9	3.2	0.08
Tumor distance to optic disc (mm),	9.9	4.2	0.02
Retina ≥ 30CGE (%)	32.7	44.1	0.02
Lens ≥ 30CGE (%)	17.0	19.7	0.87
Ciliary ≥ 30CGE (%)	30.52	30.24	0.73
Macula≥ 30CGE (%)	39.3	82.5	0.01
Optic disc≥ 12CGE (%)	18.4	78.6	0.01
Optic disc≥ 30CGE (%)	14.28	72.4	0.01

* The Mann-Whitney U-test was used to compare the two groups. A *P* value <0.05 was considered to indicate statistical significance.

According to correlation analysis, the parameters which showed the significant differences between two groups (Table 2) also showed the statistically significant correlation with final BCVA (Table 3). Most factors showing statistically significant differences were correlated negatively with the final BCVA. The distance from the tumor margin to the disc distance was correlated positively with the final BCVA; however, the distance from the tumor margin to the macula was not correlated with the final BCVA. Using the univariate regression analysis, the factors showing a statistically significant prediction of the BCVA in correlation analysis also showed the statistically significant prediction of the BCVA (Table 4), however in the multivariate regression analysis, there was no factors that showed a statistically significant prediction of the BCVA.

**Table 3:** Correlations between final BCVA and the factors expected to affect final BCVA in 28 patients who had pretreatment BCVA (≥20/40).

	Final BCVA	
	*r*	p*
Age	-0.040	0.84
Tumor height (mm),	-0.479	0.01
Largest tumor diameter (mm),	-0.451	0.02
Tumor distance to fovea (mm),	0.336	0.08
Tumor distance to optic disc (mm),	0.575	0.00
Retina ≥ 30CGE (%)	-0.566	0.00
Lens ≥ 30CGE (%)	-0.031	0.38
Ciliary ≥ 30CGE (%)	0.071	0.72
Macula≥ 30CGE (%)	-0.49	0.01
Optic disc≥ 12CGE (%)	-0.626	0.00
Optic disc≥ 30CGE (%)	-0.634	0.00

BCVA = best corrected visual acuity, CGE = cobalt gray equivalents.

* Pearson’s correlation analysis.

**Table 4:** Univariate regression analysis between final BCVA and the factors expected to affect final BCVA in 28 patients who had pretreatment BCVA (≥20/40).

	Final BCVA	
	B	p*
Age	-0.001	0.850
Tumor height (mm),	-0.090	0.020
Largest tumor diameter (mm),	-0.071	0.010
Tumor distance to fovea (mm),	0.043	0.025
Tumor distance to optic disc (mm),	0.048	0.004
Retina ≥ 30CGE (%)	-0.031	0.001
Lens ≥ 30CGE (%)	-0.002	0.742
Ciliary ≥ 30CGE (%)	0.001	0.940
Macula≥ 30CGE (%)	-0.005	0.012
Optic disc≥ 12CGE (%)	-0.006	0.001
Optic disc≥ 30CGE (%)	-0.006	0.001

BCVA = best corrected visual acuity, CGE = cobalt gray equivalents.

**Fig 2:**
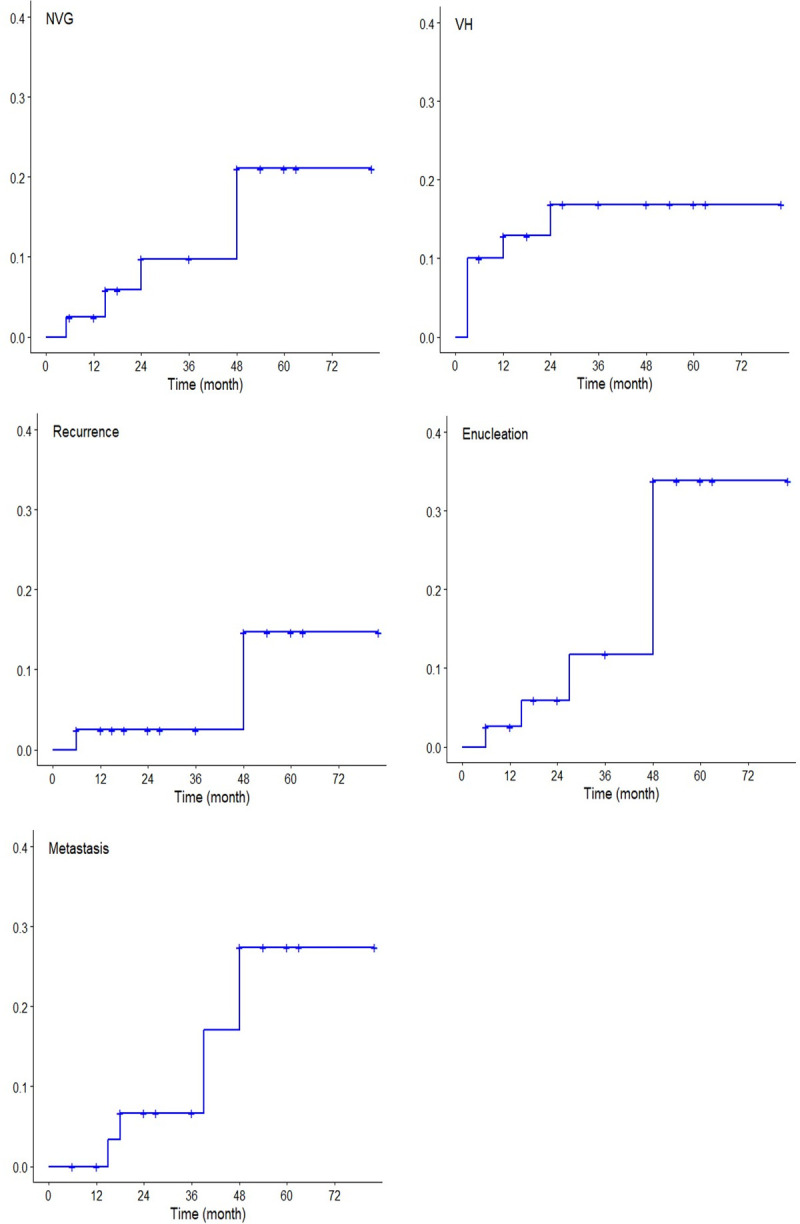
Kaplan-Meier Curve of ocular effects after Proton Beam Therapy for all choroidal melanoma patients. a) the cumulative incidence rate of neovascular glaucoma b) the cumulative incidence of vitreous hemorrhage c) the cumulative incidence of local recurrence d) the cumulative incidence of enucleation e) the cumulative incidence of distant metastasis.

A total of 40 patients, 12 patients finally showed a poor VA (< 20/200) during the follow-up period. The causes of the poor VA within 6 months were vitreous hemorrhage (four cases), followed by retinal detachment (three cases), and radiation-induced optic neuropathy (three cases). We experienced 2 cases of local recurrences, 4 cases of enucleation and 4 cases of distant metastasis. We analyzed the ocular effect of PBT using the Kaplan-Meier estimates to show the occurrence timing and frequency (Table 5, Fig 2). Fig 2 showed the cumulative incidence rate as time passed. The cumulative incidence of vitreous hemorrhage was 12.9% in 1-year and 16.9% in 5– year. On the other hand, that of neovascular glaucoma was 2.5% in 1-year and increased to 21.1% in 5- year. Vitreous hemorrhage tended to occur initially and neovascular glaucoma tended to occur relatively later.

**Table 5:** Kaplan-Meier Analysis of outcomes of Proton Bean Therapy for choroidal melanoma (N = 40).

	Kaplan-Meier Estimates, Cumulative incidence rate [95%CI]		
Outcomes	1-year	3-year	5- year
Ocular complications			
Neovascular glaucoma	0.025[0–0.072]	0.098[0–0.199]	0.211[0–0.408]
Vitreous hemorrhage	0.129[0.016–0.229]	0.169[0.032–0.286]	0.169[0.032–0.286]
Local recurrences	0.025[0–0.072]	0.025[0–0.072]	0.147[0–0.347]
Enucleation	0.026[0–0.074]	0.118[0–0.243]	0.339[0–0.569]
Distant metastasis	-	0.067 [0–0.152]	0.274[0–0.050]

## Discussion

PBT is the well-accepted treatment of choice for local tumor control and eyeball preservation [9, 10]. The ideal goal of PBT is to minimize the ocular side effects and to improve visual outcomes, in addition to local tumor control. For these reasons, an understanding of long-term visual outcomes and their associations with radiation treatment planning parameters are critical for optimal care of choroidal melanoma patients. There have been several reports on visual outcomes after radiation therapy, as well as on factors associated with visual outcomes [14, 17–19]. It is well known that tumor height and distance of the tumor from the optic disc or fovea are commonly associated with the final VA [18–20]. These studies had many patients, and long follow-up periods; therefore, they showed similar results.

In the present study, we also wanted to characterize the visual outcomes after PBT, analyze the factors affecting the final BCVA, and make a comparison with results from previous studies. As mentioned earlier, choroidal melanoma is very rare in Asian countries and there has been no study of visual outcomes in Asian patients. We included only 40 patients, so we could not classify them according to the size or tumor locations. Instead, we used dose-volume histogram (DVH) analysis.

A DVH is a histogram relating radiation dose to tissue volume in radiation therapy planning [21]. DVHs are most commonly used as a plan evaluation tool and to compare doses from different plans. The DVH summarizes three-dimensional (3D) dose distributions in a graphical two-dimensional format. In modern radiation therapy, 3D dose distributions are typically created in a computerized treatment planning system based on a 3D reconstruction of a CT scan. The vertical axis is almost always plotted as percent volume (rather than absolute volume). We used cumulative DVH, with the retina ≥ 30 CGE (%), which was the proportion of the retinal area receiving ≥ 30 CGEs (proton relative biological effectiveness = 1.1). A previous study including 645 patients reported that 28 GyE exposure of the macula and optic nerve was an independent DVH predictor of post-PRBT vision loss among those initially with a favorable VA [13]. In the present study, we also analyzed the correlation between the final BCVA and the proton radiation dose and, in particular, subdivided the dose of proton radiation in the optic disc area as the same or > 12 CGE or 30 CGE. The results of this showed similar results to the previous study; the amount of radiation in the optic disc and macula area were strongly associated with the final visual outcomes. We tried to perform multivariable regression analysis additionally between the final visual acuity and previous mentioned parameters. However, we could not find the statistical significant association. It was thought that we had small number of patients in this study and there were parameters which could affect one another. The radiation dose in the anterior portion of the eyeball, such as the lens or ciliary body, did not significantly affect the final BCVA. It is well known that DVH metrics correlate with patient toxicity outcomes [22]. If the radiation dose increases, the possibility of radiation-induced cataract or injury in the trabecular meshwork would be expected to increase. However, the influence on the final BCVA by anterior segment damage would be predicted to be insignificant, and other reports showed its effect was not higher than that of the posterior segment, such as the optic disc or macula [23].

Regarding visual outcomes, long-term visual outcomes after radiation therapy have often been reported. Urie et al., in 1986, reported that prognostic factors for visual loss following proton irradiation in 440 eyes were tumor height, distance of the tumor from the optic disc and fovea, worse pretreatment vision, and high radiation doses delivered to both the disc and fovea, or lens [20]. Since that report, there have been various reports including visual outcomes and ocular side effects after radiation therapy using protons or plaques. During the follow-up period, we wondered when a rapid VA decrease occurred, and when this decline entered the static phase. For these reasons, we drew the trend line of the VA in choroidal melanoma patients, and found most patients experienced rapid VA decreases within 6 months after PBT, while the changes of VA were minimal after 12 months (Fig 1). Several studies including many patients and long-term follow-up periods showed that the majority of changes in the BCVA occurred within the first 24‒36 months of follow-up, with relative stability seen afterwards [13, 24–27]. The differences seem to be caused by cases in our study involving initial short-term intervals, with an overall short follow-up period. Further studies are therefore needed.

It is well known that radiation induces ocular side effects, such as hemorrhage, exudate, macular edema, and nerve fiber infarction, finally resulting in visual loss [12]. It was surprising to find that the causes of visual loss were different in the early and late phases after PBT. In the group with a pretreatment BCVA of < 20/40, there was already a huge tumor mass, which sometimes disturbed the visual axis, usually resulting in rapid visual loss (Fig 1B). In the group with a pretreatment BCVA of ≥ 20/40, the changes of BCVA varied according to the tumor location or size. Some patients experienced rapid VA loss and others experienced gradual VA loss. There were some patients who initially experienced vitreous hemorrhage or retinal detachment after PBT, resulting in permanent visual loss. A few cases had an excellent VA during the initial 12 months, then suddenly experienced a rapid VA decrease with neovascularization of the iris or retina. The most important ocular complications in this study involved neovascular glaucoma (NVG). Vitreous hemorrhage or retinal detachment mostly occurred in the group who had a pretreatment BCVA of < 20/40; therefore, the change of ocular discomfort in patients was minimal. However, in cases of neovascular glaucoma, most patients were tolerable, meaning that they had a BCVA of ≥ 20/200 after PBT and experienced abrupt ocular pain or decreasing VA unexpectedly. Based on previous studies, NVG patients had significant treatment complications and the 5-year NVG incidence was up to 35%; most post-radiation enucleations were related to the NVG [23, 28]. *Damato et al* reported the similar results with our study in the choroidal melanoma patients after Ru-106 brachytherapy and explained several possible mechanisms for visual loss [29]. They reported that visual loss before treatment correlated with poor visual outcome. Such reduced initial visual acuity indicated direct macular involvement by tumor or exudate. Also posterior tumor extension was associated with visual loss after treatment, and this had several possible mechanisms including immediate radiation damage to macula or optic disc and late damage due to the release of vasoproliferative factors.

There were several limitations to this study. This was a retrospective study, and we included a small number of patients and relatively short follow-up periods, compared with the other studies in Western countries. Nevertheless, this study produced novel results, which included the effect of PBT on choroidal melanoma in patients in Asia, and was the first report emphasizing the visual outcomes of PBT in Asia.

In conclusion, approximately one-third of PBT-treated choroidal melanoma patients with good pretreatment BCVA maintained their VA. Rapid visual decline occurred in patients who finally had poor visual outcomes after 6‒12 months of PBT. Tumor size, tumor distance to the optic disc or fovea, volume of the macula and optic disc or retina receiving radiation would be expected to affect the final VA.
